# Efficacy of at home monitoring of foot temperature for risk reduction of diabetes‐related foot ulcer: A meta‐analysis

**DOI:** 10.1002/dmrr.3549

**Published:** 2022-06-08

**Authors:** Jonathan Golledge, Malindu E Fernando, Chanika Alahakoon, Peter A. Lazzarini, Wouter B. aan de Stegge, Jaap J. van Netten, Sicco A. Bus

**Affiliations:** ^1^ Ulcer and wound Healing consortium (UHEAL) Queensland Research Centre for Peripheral Vascular Disease College of Medicine and Dentistry James Cook University Townsville Queensland Australia; ^2^ Department of Vascular and Endovascular Surgery Townsville University Hospital Townsville Queensland Australia; ^3^ Australian Institute of Tropical Health and Medicine James Cook University Townsville Queensland Australia; ^4^ School of Public Health and Social Work Queensland University of Technology Brisbane Queensland Australia; ^5^ Allied Health Research Collaborative Metro North Hospital and Health Service Brisbane Queensland Australia; ^6^ Amsterdam UMC, University of Amsterdam Department of Rehabilitation Medicine Amsterdam Movement Sciences Amsterdam The Netherlands; ^7^ Department of Surgery University of Groningen Groningen The Netherlands

**Keywords:** diabetic foot ulcers, secondary prevention, temperature monitoring

## Abstract

**Aims:**

To perform an updated systematic review of randomised controlled trials examining the efficacy of at‐home foot temperature monitoring in reducing the risk of a diabetes‐related foot ulcer (DFU).

**Methods:**

Systematic review performed according to Preferred Reporting Items for Systematic reviews and Meta‐Analyses guidelines. Risk‐of‐bias was assessed using version 2 of the Cochrane risk‐of‐bias tool. Meta‐analyses were performed using random effect models. Leave‐one‐out sensitivity analyses and a sub‐analysis excluding trials considered at high risk‐of‐bias assessed the consistency of the findings. The certainty of the evidence was assessed with GRADE.

**Results:**

Five randomised controlled trials involving 772 participants meeting the International Working Group on the Diabetic Foot (IWGDF) risk category 2 or 3 were included. All trials reported instructing participants to measure skin temperature at‐home at six or more sites on each foot using a hand‐held infra‐red thermometer at least daily and reduce ambulatory activity in response to hotspots (temperature differences >2.2°C on two consecutive days between similar locations in both feet). One, one, and three trials were considered at low, moderate and high risk‐of‐bias, respectively. Participants allocated to at‐home foot temperature monitoring had a reduced risk of developing a DFU (relative risk 0.51, 95% CI 0.31–0.84) compared to controls. Sensitivity and sub‐analyses suggested that the significance of this finding was consistent. The GRADE assessment suggested a low degree of certainty in the finding.

**Conclusions:**

At‐home daily foot temperature monitoring and reduction of ambulatory activity in response to hotspots reduce the risk of a DFU in moderate or high risk people with a low level of certainty.

## 1 INTRODUCTION

Diabetes‐related foot ulcers are a leading cause of disability, hospital admission, and healthcare costs.^1^ Most diabetes‐related foot ulcers develop due to the cumulative effects of high plantar pressures and ambulatory activity in people with insensate feet secondary to peripheral neuropathy.^2^ Warning signs of repetitive trauma to the soles of feet can be identified by raised skin temperature at a site on one foot compared to the similar site on the contralateral foot, termed a hotspot.^3^ Such focal hotspots are believed to represent areas of inflammation resulting from repetitive trauma and have been shown to predict the development of ulcers at that site.^3^ Once a hotspot is identified, offloading of the relevant area, usually through reduction of ambulatory activity and/or reduction of plantar pressures via footwear modifications, can help prevent ulcer development.^4^

A number of randomised controlled trials have examined the effect of at‐home foot temperature monitoring and reduction of ambulatory activity in response to hotspots on the risk of developing a diabetes‐related foot ulcer.^4^, ^5^, ^6^, ^7^, ^8^ Two older trials mainly included participants who had no prior history of foot ulceration and were considered at moderate risk of foot ulcer formation (International Working Group on the Diabetic Foot [IWGDF] risk categories 2).^7^, ^8^ Whereas, three more recent trials mainly included participants who had a previous foot ulcer and were considered at high risk of new ulcer formation (IWGDF risk categories 3) and thus more likely to benefit from such preventative care.^4^, ^5^, ^6^

Recent meta‐analyses of randomised controlled trials suggest that at‐home foot temperature monitoring and reduction of ambulatory activity in response to hotspots reduce the risk of developing a diabetes‐related foot ulcer in moderate or high risk participants.^9^, ^10^ The significance of this finding was, however, not consistent in meta‐analyses when a leave‐one‐out sensitivity analysis was performed^9^ and the overall interpretation was limited by the relatively small pooled sample size of the included trials (*n* = 468).^9^, ^10^ However, since the publication of these recent meta‐analyses, a much larger trial (*n* = 304) of participants considered at high risk of new ulcers has been completed, enabling nearly a doubling of the pooled sample size of previous meta‐analyses.^5^ Furthermore, since the previous meta‐analyses, Cochrane has published version 2 of the Cochrane risk‐of‐bias tool, also enabling a much more contemporary assessment of risk of bias than previous meta‐analyses. There is therefore a need for an updated meta‐analysis to clarify the pooled evidence of benefit for at‐home foot temperature monitoring and reduction of ambulatory activity in response to hotspots in preventing a diabetes‐related foot ulcer. The aim of this study was to perform a systematic review and meta‐analysis of randomised controlled trials testing the efficacy of at‐home foot temperature monitoring and reduction of ambulatory activity in response to hotspots on reducing the risk of a diabetes‐related foot ulcer.

## 2 MATERIAL AND METHODS

### 2.1 Search strategy and eligibility criteria

This systematic review was conducted according to the Preferred Reporting Items for Systematic reviews and Meta‐Analyses (PRISMA) statement and registered in the PROSPERO database (Registration number: 235955).^11^ The Medline (via OvidSP, 1966), PubMed, Web of Science (via ISI Web of Knowledge; 1965), and The Cochrane Library databases were searched from inception to 14 July 2021. The search terms used are detailed in the Supplement. No language or date restrictions were used. Reference lists of the studies identified were also searched. Eligibility criteria for inclusion were: A randomised controlled trial testing of at‐home foot temperature monitoring and reduction of ambulatory activity in response to hotspots; inclusion of a control group not receiving at‐home foot temperature monitoring but otherwise receiving similar care; that the study included participants that had diabetes and were at risk of developing diabetes‐related foot ulcers (defined as IWGDF risk categories 2 or 3^12^); and the incidence of foot ulcers during follow‐up was reported. Studies including participants with a diabetes‐related foot ulcer were excluded. At‐home foot temperature monitoring was defined as the assessment of foot skin temperature by the participants using an objective temperature monitoring device at home. Diabetes‐related foot ulcer was defined as a full thickness wound on the foot of a person with diabetes.^13^

### 2.2 Data extraction

The primary outcome was the development of any diabetes‐related foot ulcer during follow‐up. Secondary outcomes were minor and major amputations. Other outcomes collected were adherence to foot temperature monitoring, frequency of contacting the study nurse or podiatrist, and amount of ambulatory activity reductions in response to hotspots. Outcome data were extracted for the latest time point reported. Other data extracted included age, sex, body mass index (BMI), duration of diabetes, glycosylated haemoglobin levels (HbA1C), ankle‐brachial pressure index (ABPI), and loss to follow‐up.^14^ Loss to follow‐up was defined as participants in which primary outcome data were not reported by the completion of follow‐up. Data were extracted by three authors separately and inconsistencies were resolved through discussion.

### 2.3 Quality assessment

Risk of bias of each included trial was assessed independently by two of three authors (PAL, JJV, and CA) using version 2 of the Cochrane risk‐of‐bias tool for randomised controlled trials.^15^ Total risk of bias for each study was then defined as: low risk: if low risk of bias was scored for each of the five elements of the risk‐of‐bias assessment; moderate risk: if some concerns (but no high risks) were scored in assessments of one or two (<50%) of the five elements of the risk‐of‐bias assessment; high risk: if high risk of bias was scored on one or more elements or some concerns were scored on three or more elements.^15^ Any inconsistencies were resolved through discussion until a consensus was reached.

### 2.4 Data analysis

Meta‐analyses were planned to be performed for any of the primary and secondary outcomes if data were reported in at least three trials. A sub‐analysis was also planned to exclude any studies deemed to be at high risk of bias.^15^ All meta‐analyses were performed using Mantel‐Haenszel's statistical method and random effect models anticipating substantial heterogeneity.^16^ The results were reported as relative risk (RR) and 95% confidence intervals (CI). All meta‐analyses assumed that participants lost to follow‐up did not have outcome events (best case scenario). All statistical tests were two‐sided and *p*‐values <0.05 were considered significant. Statistical heterogeneity was assessed using the *I* ^2^ statistic and interpreted as low (0%–49%), moderate (50%–74%), or high (75%–100%).^17^ Leave‐one‐out‐sensitivity analyses were performed to assess the contribution of each study to the pooled estimates by excluding individual studies one at a time and recalculating the pooled estimates.^18^ Publication bias was assessed by funnel plots comparing the summary estimate of each study and its precision (1/standard error).^18^ All analyses were conducted using Review Manager 5 (RevMan 5) version 5.4. (Copenhagen: Nordic Cochrane Centre, The Cochrane Collaboration, 2014).

### 2.5 Certainty of the evidence assessment

The overall certainty (quality) of the evidence was assessed according to the Grading of Recommendations Assessment, Development and Evaluation (GRADE) using the GRADEpro guideline development tool (GDT) (https://gradepro.org/) to evaluate the risk of bias, inconsistency, indirectness, and imprecision of the combined trial evidence.^19^

## 3 RESULTS

### 3.1 Included trials and participants

**FIGURE 1:**
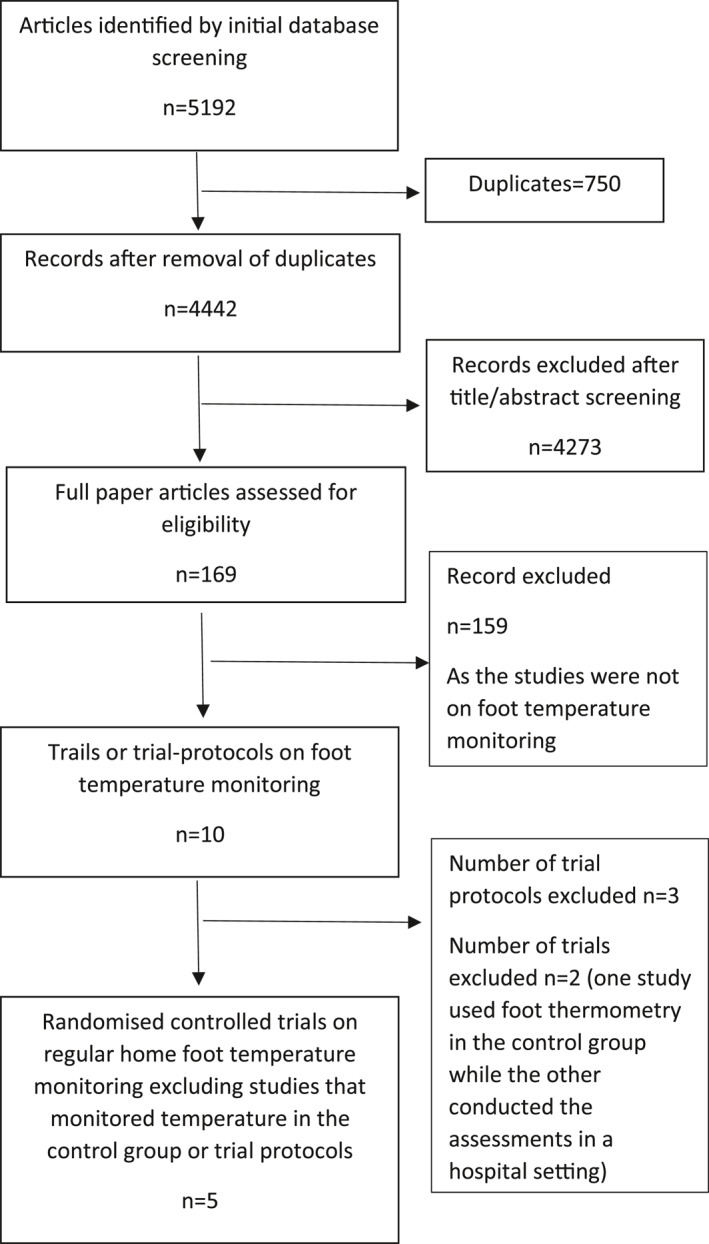
Preferred Reporting Items for Systematic reviews and Meta‐Analyses diagram illustrating the identification and selection of the included studies

A total of 5192 articles were identified from the initial search and ultimately, 5 trials were included (Figure 1). A total of 772 participants of IWGDF risk categories 2 and 3 were included in the five trials (Table 1). The trials were conducted in the Netherlands,^5^ Norway,^6^ and USA.^4^, ^7^, ^8^ Details of the inclusion criteria, interventions, controls, and outcome measures are shown in Supplementary Information S1. All trials used a similar infrared thermometer (TempTouch, Xilas Medical, San Antonio, Texas) for participants to monitor the at‐home skin temperature at six or more sites on each foot either once^7^, ^8^ or twice^4^, ^5^, ^6^ daily (Supplementary Information S1). All studies informed the participants to reduce ambulatory activity and contact a study nurse or podiatrist if they observed a temperature difference of ≥2.2°C between corresponding regions in the left and right foot for two consecutive days, defined as a hotspot. Two trials indicated to participants that ambulatory activity should be reduced by half when identifying a hotspot,^5^, ^6^ while the amount of ambulatory activity reduction to be undertaken was not reported in the other three trials.^4^, ^7^, ^8^ Four trials instructed the participants to reduce ambulatory activity taken during the following days until the temperature difference was <2.2°C.^5^, ^6^, ^7^, ^8^ All intervention and control groups had access to therapeutic footwear, diabetic foot education, and regular foot care (Supplementary Information S1).

**TABLE 1:** Baseline characteristics of participants

Study	Group	Number of patients randomised	Age (years)	Male %	Follow up (months)	Previous history of DFU %	Previous history of amputations %	Duration of DM in years	HbA1c %
Lavery et al. (2004)^13^	Intervention	41^b^	55.0 ± 9.3	49	6	41	2	14.8 ± 11.5	NR
	Control	44^b^	54.8 ± 9.6	52	6	41	2	12.7 ± 10.0	NR
Armstrong et al. (2007)^12^	Intervention	111	68.2 ± 9.6	98	18	15	NR	13.6 ± 11.6	8.1 ± 1.9
	Control	114	69.7 ± 10.4	95	18	17	NR	12.6 ± 9.1	7.4 ± 1.4
Lavery et al. (2007)^15^	Intervention	59	65.4 ± 9.3	56	15	100	22	12.7 ± 9.7	NR
	Control	58	65.0 ± 9.6	53	15	100	31	13.7 ± 10.3	NR
Skafjeld et al. (2015)^14^	Intervention	21	57.1 ± 10.2	86	12	100	33^c^	17.0 (NR)^a^	8.3 ± 1.5
	Control	20	59.4 ± 13.0	75	12	100	40^c^	19.5 (NR)^a^	7.9 ± 1.7
Bus et al. (2021)^5^	Intervention	151	65.0 ± 10.6	72	18	97	27	20 ± 14	7.7 ± 3.7
	Control	153	64.2 ± 10.5	73	18	97	26	21 ± 15	7.7 ± 3.6

*Note*: Data is shown as numbers or mean ± standard deviation or percentages unless otherwise highlighted. To convert percentage HbA1c values to mmol HbA1c per mol Hb use the following equation 10.93 × % hbA1c − 23.5 mmol/mol.

Abbreviations: DFU, diabetes‐associated foot ulcer; DM, diabetes mellitus; HbA1c, glycated haemoglobin; NR, not reported.

^a^ Data were reported as median (inter quartile range).

^b^ Based on the data presented in results in‐contrast to the numbers presented in the abstract.

^c^ Included participants with a history of toe amputations.

### 3.2 Risk of bias

Table 2 and Supplementary Information S1 display the findings of the quality assessment. One trial was deemed to have a low risk of bias,^5^ one a moderate risk of bias,^6^ and three a high risk of bias.^4^, ^7^, ^8^ Some concerns or high risk of bias were identified in the assessment of four of the five elements, namely the randomisation process in one trial, 8 deviation from the intended intervention in two trials,^4^, ^7^ and both measurement of the outcome and selection of the reported results in four trials^4^, ^6^, ^7^, ^8^ (Table 2 and Supplementary Information S1).

**TABLE 2:** Summary table of the quality assessment

Study	Risk of bias element assessed					Overall risk of bias
	Randomisation process	Deviation from intended intervention	Missing outcome data	Measurement of outcome	Selection of reported results	
Lavery et al. (2004)^13^	(±)	(+)	(+)	(−)	(±)	High
Armstrong et al. (2007)^12^	(+)	(±)	(+)	(±)	(±)	High
Lavery et al. (2007)^15^	(+)	(±)	(+)	(−)	(±)	High
Skafjeld et al. (2015)^14^	(+)	(+)	(+)	(±)	(±)	Moderate
Bus et al. (2021)^5^	(+)	(+)	(+)	(+)	(+)	Low

*Note*: (+) Low; (±) Some concerns; (−) High. In the overall risk of bias, some concerns were interpreted as moderate risk of bias.

### 3.3 Effect of at‐home temperature monitoring on foot ulcer risk in individual trials

The primary outcome in four trials was developing a diabetes‐related foot ulcer at any site^4^, ^6^, ^7^, ^8^ and in the most recent trial, this was a secondary outcome.^5^ Overall, four of the five trials reported that the intervention significantly reduced the risk of developing a diabetes‐related foot ulcer at any site compared to the control after 6–18 months^4^, ^5^, ^7^, ^8^ (Table 3). In the most recent trial, the primary outcome was a foot ulcer that developed at, or adjacent to, a site where temperature measurements were performed and for this outcome, there was no significant difference between groups (44 of 151 in intervention vs. 57 of 153 in control; *p* = 0.133). Only two trials reported amputation outcomes with no significant difference between groups (Table 3).^5^, ^8^

**TABLE 3:** Outcome data from individual studies

Study		Participants developing a foot ulcer (reported per number initially randomised)	Incidence of major amputations	Incidence of minor amputations	Incidence of hotspots	Study nurse or podiatrist contact	Reduced ambulatory activity in response to hotspot	Loss to follow up
Lavery et al. (2004)^13^	Intervention	1/41 (2.4%)^a^	0/41 (0.0%)	0/41 (0.0%)	NR	NR	NR	3
	Control	7/44 (15.9%)^a^	0/44 (0.0%)	0/44 (0.0%)	NA	NR	NA	4
Armstrong et al. (2007)^12^	Intervention	5/111 (4.5%)^a^	NR	NR	NR	NR	NR	NR
	Control	14/114 (12.2%)^a^	NR	NR	NA	NR	NA	NR
Lavery et al. (2007)^15^	Intervention	5/59 (8.5%)^a^	NR	NR	38/59 (64.4%)	31/59^a^ (53.0%)	NR	6
	Control	17/58 (29.3%)^a^	NR	NR	NA	18/58 (31.0%)	NA	3
Skafjeld et al. (2015)^14^	Intervention	7/21 (33.3%)	NR	NR	8/21 (38.0%)	10/21 (48.0%)	NR	3
	Control	10/20 (50.0%)	NR	NR	NA	12/20 (60.0%)	NA	0
Bus et al. (2021)^5^	Intervention	54/151 (35.8%)^a^	1/151 (0.7%)	3/151 (2.0%)	83/151 (55.0%)	14/151 (9.3%)	24/83 (28.9%)	0
	Control	72/153 (47.1%)^a^	1/153 (0.7%)	6/153 (3.9%)	NA	NR	NA	0

Abbreviations: DFU, diabetes‐related foot ulcer; NA: not applicable; NR, not reported.

^a^ Indicates studies with significant differences between the intervention and the control groups.

### 3.4 Meta‐analyses

**FIGURE 2:**
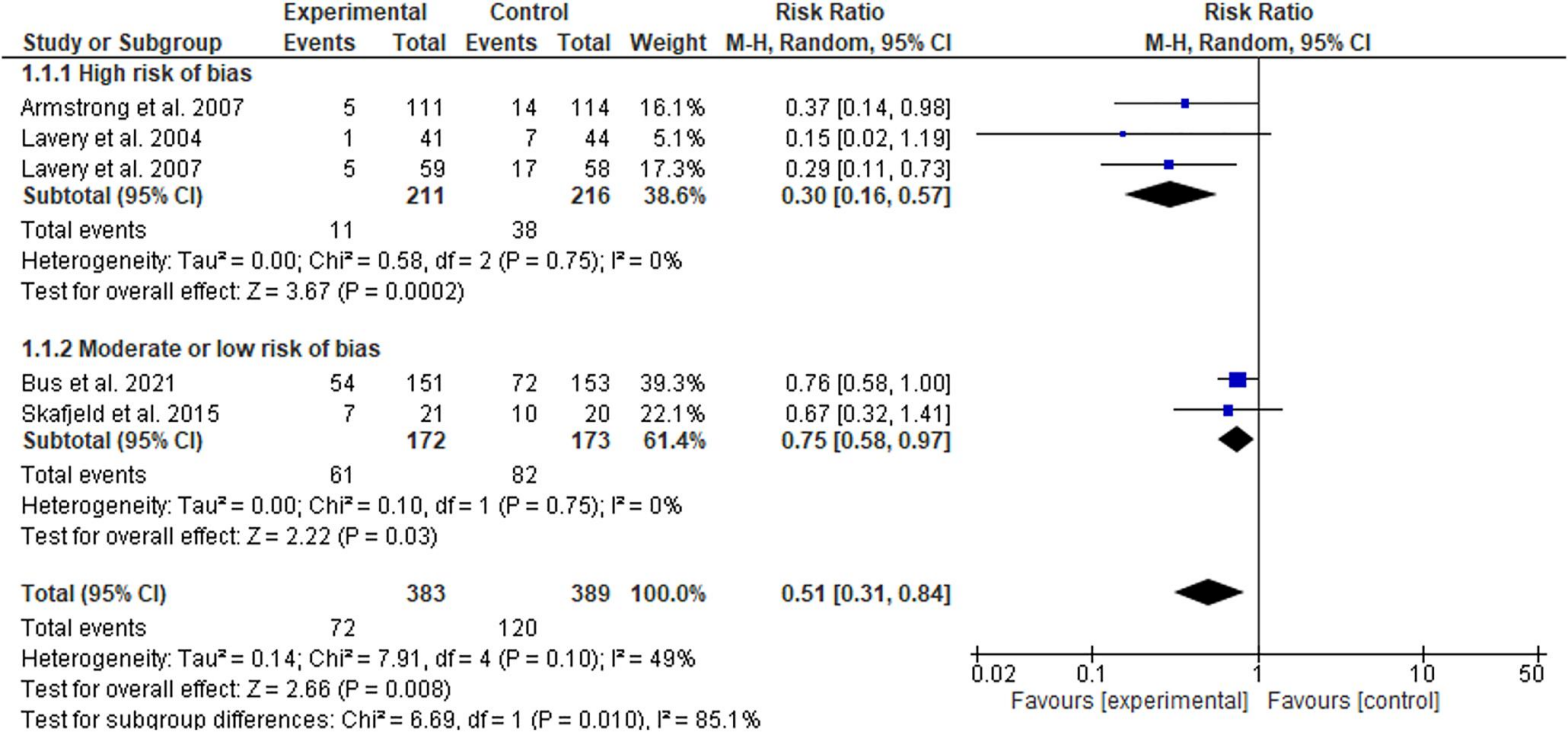
Effect of at‐home foot temperature monitoring with infrared thermometry and offloading of hotspots in prevention of diabetes‐related foot ulcers

**FIGURE 3:**
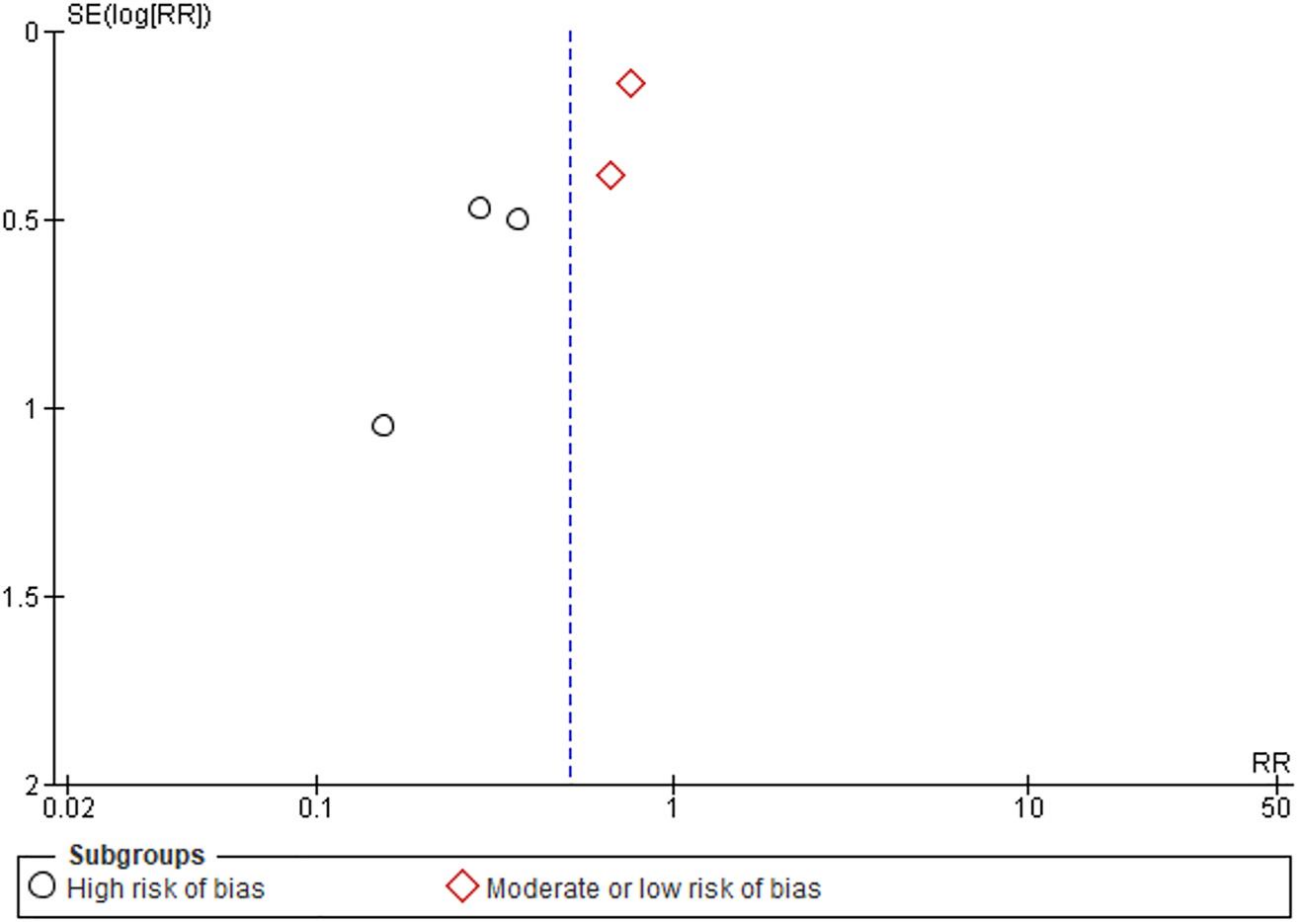
Funnel plot assessing likelihood of publication bias

The meta‐analysis suggested that at‐home foot temperature monitoring and reduction of ambulatory activity in response to hotspots halved the risk of developing any diabetes‐related foot ulcer (RR 0.51, 95% CI 0.31–0.84, *n* = 772), with low statistical heterogeneity between studies (*I* ^2^ = 49%) (Figure 2). Leave‐one‐out sensitivity analyses showed that the significance of the findings were independent of the inclusion of any single trial (Table 4). The statistical heterogeneity however, as assessed by *I* ^2^, decreased to 10% after the exclusion of the trial reported by Bus et al.^5^ and increased to 62% after excluding the trial reported by Skafjeld et al.^6^ (Table 4). In the sub‐analysis, the three trials assessed as having a high risk of bias^4^, ^7^, ^8^ and the two trials assessed as having a moderate^6^ or low risk of bias^5^ were separately analysed. The overall RR reduction in the high risk of bias trials was greater (0.30, 95% CI 0.16–0.57) than in the lower risk of bias trials (0.75, 95% CI 0.58–0.97) (Figure 2). The funnel plot was symmetrical suggesting a low likelihood of publication bias (Figure 3). A meta‐analysis focussed on amputations was not possible due to lack of sufficient trials that reported these outcomes.

**TABLE 4:** Leave‐one‐out sensitivity analyses

Excluded study	Relative risk	Heterogeneity
None	0.51 [0.31, 0.84]	Heterogeneity: Tau^2^ = 0.14; Chi^2^ = 7.91, df = 4 (*p* = 0.10); *I* ^2^ = 49%
Lavery et al. (2004)^13^	0.56 [0.35, 0.89]	Heterogeneity: Tau^2^ = 0.10; Chi^2^ = 5.68, df = 3 (*p* = 0.13); *I* ^2^ = 47%
Armstrong et al. (2007)^12^	0.54 [0.31, 0.94]	Heterogeneity: Tau^2^ = 0.16; Chi^2^ = 6.28, df = 3 (*p* = 0.10); *I* ^2^ = 52%
Lavery et al. (2007)^15^	0.62 [0.40, 0.95]	Heterogeneity: Tau^2^ = 0.07; Chi^2^ = 4.37, df = 3 (*p* = 0.22); *I* ^2^ = 31%
Skafjeld et al. (2015)^14^	0.43 [0.21, 0.88]	Heterogeneity: Tau^2^ = 0.30; Chi^2^ = 7.95, df = 3 (*p* = 0.05); *I* ^2^ = 62%
Bus et al. (2021)^5^	0.41 [0.25, 0.70]	Heterogeneity: Tau^2^ = 0.03; Chi^2^ = 3.33, df = 3 (*p* = 0.34); *I* ^2^ = 10%

### 3.5 GRADE assessment of the evidence

A summary of the GRADE certainty (quality) of the evidence assessment is provided in Table 5. The risk‐of‐bias item was rated as serious due to three of the five trials being assessed as at high risk of bias. Inconsistency was also rated as serious due to the variation in point estimates noted for the three trials considered to be at a higher risk of bias compared to the two trials considered to be at a lower risk of bias (Figure 2). Indirectness was deemed as not serious since all included trials tested the effect of the same intervention. Imprecision was rated as not serious as it was deemed that the optimal information size was met, based on a sample size estimate that tested a relative risk for the intervention of 0.70, an event rate of 40%, power of 90%, alpha of 0.05, and drop‐out rate of 8%, which was lower (*n* = 764) than the total included patients (*n* = 772).

**TABLE 5:** GRADE Certainty of the evidence assessment for at‐home temperature monitoring on incidence of diabetes‐related foot ulcers

Evidence assessment items					
**Studies**	**Design**	**Risk of bias**	**Inconsistency**	**Indirectness**	**Imprecision**
5	Randomised trials	Serious^a^	Serious^b^	Not serious	Not serious

**Summary of findings**					
**Number of events**		**Effect**			
**Temperature monitoring**	**Control**	**RR (95% CI)**	**Absolute (95% CI)**	**Certainty (quality) of evidence**	
72/383 (18.8%)	120/389 (30.8%)	0.51 (0.31–0.84)	151 fewer events per 1000	Low	

Abbreviations: CI, Confidence intervals; GRADE, Grading of Recommendations, Assessment, Development and Evaluation; RR, relative risk.

^a^ Downgraded from high to moderate certainty of evidence due to three of the five trials assessed as at high risk of bias.

^b^ Further downgraded from moderate to low certainty of evidence due to variations in point estimates for the three at high risk of bias trials compared to the two trials deemed to be at lower risk of bias.

### 3.6 Adherence to the intervention and association with outcome

Adherence to at‐home foot temperature monitoring by intervention participants was reported in variable ways in three trials^4^, ^5^, ^6^ and not at all in two trials^7^, ^8^ (Supplementary Information S1). Lavery et al. 2007 reported monitoring adherence to the intervention, but provided no details of how this was assessed.^4^ They reported that 38 of the 59 (64.4%) participants in the intervention group detected hotspots over 15 months and decreased their ambulatory activity in response by a mean (±standard deviation) of 1725 ± 1784 steps/day.^4^ Also, significantly more participants in the intervention than in the control group contacted the study nurse due to concerns about their feet (53.0% vs. 31.0%, Table 3) and significantly less developed ulcers (8.5% vs. 29.3%, Table 3). Participants within the intervention group that measured foot temperature >50% of the required days were also reported to be significantly less likely to develop a foot ulcer compared to participants measuring foot temperature <50% of the time.^4^ Amongst participants in the intervention group, in those that developed a foot ulcer, 80% did not adhere to the temperature assessment, while in those that did develop an ulcer, 92% adhered to the temperature assessment >50% of the time. Skafjeld et al. reported monitoring adherence using a participant reported semi‐quantitative scale every 3 months.^6^ Fourteen (66.7%) participants reported they measured foot temperature ≥80% of the required days. Eight (38.1%) participants in the intervention group identified a hotspot over the 12 month follow‐up. Similar numbers of participants in the intervention and control groups contacted the study nurse (Table 3). There were no reports on whether participants reduced their ambulatory activity and to what extent. Bus et al.^5^ reported monitoring adherence via asking participants to record their temperatures in a logbook and return this to the investigators every 4 weeks. Participants received a text message to remind them to measure their foot temperature and decrease their weight‐bearing activity if a hotspot was identified. Ninety‐four (62.3%) participants recorded the foot temperature in their logbook ≥70% of the required days and in the 24 who reduced their activity by ≥50% in response to a hotspot, three (12.5%) developed a foot ulcer at or near a measurement site. This was significantly less than the 21 (35.6%) of the 59 participants who identified a hotspot but did not report to have reduced their activity.

## 4 DISCUSSION

This updated meta‐analysis suggests that the daily at‐home foot temperature monitoring and activity reduction in response to hotspots significantly reduce the risk of a diabetes‐related foot ulcer amongst people at moderate or high risk (IWGDF risk category 2 or 3). This meta‐analysis included 304 more participants than prior meta‐analyses, almost doubling the numbers previously included.^9^, ^10^ Unlike prior analyses, the findings in this updated analysis remained significant in leave‐one‐out sensitivity analyses and in a sub‐analysis excluding trials deemed at high risk of bias. The GRADE assessment of the certainty of the evidence suggested a low level of certainty due to risk of bias and inconsistency being considered serious. It should be noted though that only two trials reported including participants of IWGDF risk category 2.^7^, ^8^ Overall, this provides consistent low‐certainty evidence that at‐home foot temperature monitoring and activity reduction in response to hotspots are effective ways to reduce the risk of a diabetes‐related foot ulcer in high‐risk people.

While the overall meta‐analysis suggests this kind of intervention halves the risk of foot ulcer development, the sub‐analysis showed that the pooled RR reduction in the two trials deemed to be at a lower risk of bias was 0.75 compared to 0.30 in the three higher risk of bias trials. This finding was considered to represent a serious inconsistency using the GRADE assessment. The most recent trial did find a significant reduction in the risk of developing a foot ulcer amongst the intervention group despite having the smallest relative reduction in this outcome of all the included trials. Over time, usual preventative care as provided to control groups, such as the production and use of offloading footwear, has advanced, which may in part explain the smaller effect sizes found in more recent trials.^5^, ^6^ The most recent trials were also performed in Europe rather than USA and thus the different point estimates of effect for foot temperature monitoring could reflect distinct healthcare delivery or populations in these locations. Finally, the trials included participants with varying proportions of IWGDF risk categories 2 and 3, which may have contributed to the heterogeneity in the findings.^4^, ^5^, ^6^, ^7^, ^8^

Interestingly, all the included trials used the TempTouch (Xilas Medical, San Antonio, TX) to measure foot temperature.^5^, ^7^, ^8^, ^20^, ^21^ TempTouch is an appropriately calibrated device for measuring foot skin temperatures; however, users need to hold the device at multiple different sites on the sole of each foot and then actively record and interpret the temperatures at those sites themselves on a daily basis. This requires substantial time commitment from users, the physical and cognitive capacity to perform these daily tasks, and the flexibility to carry out this task daily over years. Such challenges may have contributed to the low adherence to monitoring temperatures that was reported in variable ways in three of the included trials.^4^, ^5^, ^6^ Where reported, better adherence, to both monitoring and activity reduction, was associated with greater efficacy of at‐home temperature monitoring in reducing the risk of foot ulcers. This suggests that temperature monitoring has to be accompanied by activity reduction in response to hotspots to be effective. The most recent trial reported the most detailed information about adherence to temperature monitoring and activity reduction.^5^ It was reported that 62% of participants measured foot temperature ≥70% of the required days but only 29% of those that identified a hotspot reported reducing their activity by ≥50%. Of these participants, very few developed an ulcer in comparison to the equivalent participants who did not report to have reduced their activity. Future research identifying temperature monitoring systems and participant support that facilitate excellent adherence is needed.

There is already a great burden of self‐monitoring placed on people with diabetes and thus a variety of forms of support may be needed for effective implementation of at‐home foot temperature monitoring. Motivational interviewing is a counselling method that has been used in a variety of populations to support healthy behaviours, such as physical activity.^22^ Such counselling methods might be a valuable means to improve adherence to at‐home foot temperature monitoring. It is also likely that easy‐to‐use devices would optimise monitoring adherence. Floor temperature mats have been developed as easy‐to‐use systems and have been reported to be associated with high rates of adherence and high validity for identifying hotspots and impending ulcer development.^3^, ^23^ These mats have been designed to provide alerts to a central team that can provide support and advice to patients. These types of mats have so far not been tested in randomised controlled trials and are currently not widely available. Thus, there is a need for further development and testing of validated, user‐friendly, and affordable methods of at‐home monitoring of foot temperature to enable effective implementation of the findings from randomised controlled trials reported in this systematic review.

Measurement of foot temperature during clinical assessments is an alternative approach to at‐home monitoring that has been suggested, although this is not feasible to perform on a daily basis. A recent clinical trial tested the benefit of such foot temperature monitoring performed at monthly intervals at an outpatient clinic.^24^ A validated thermal camera was used to identify hotspots in the clinic, which were treated by advising reductions in physical activity and improved offloading of the affected area.^24^ The trial included 110 participants with a past history of a diabetes‐related foot ulcers and reported no benefit of the intervention in preventing ulcers or improving health‐related quality of life.^24^ Thus, the findings of that trial along with this updated meta‐analysis suggest that daily at‐home monitoring of foot temperature is required for this preventative treatment to be effective.

A number of limitations of the included trials and this meta‐analysis should be acknowledged. The risk of bias of three of the included trials was considered to be high. Elements considered to be at risk of bias included the randomisation process, deviation from the intended intervention, measurement of the outcome, and selection of reported results. There was also heterogeneity in follow‐up time and in reporting patient characteristics. The GRADE assessment identified a low level of certainty in the findings. Lastly, the included trials all took place in Europe and the US, therefore the overall effectiveness of this intervention outside of these continents remains uncertain. Hence, further evaluation of temperature monitoring interventions in different climates and in different populations, particularly those at different risk for ulcers, is needed to evaluate its scalability more globally.

In conclusion, this meta‐analysis provides promising but low‐certainty evidence that daily at‐home foot temperature monitoring and reduction of activity in response to hotspots are effective at reducing the risk of a diabetes‐related foot ulcer in at‐risk people. Effective, user‐friendly, and affordable intervention systems are needed for foot temperature monitoring and the necessary thresholds of action in response to identifying hotspots, for widespread adoption of this preventative intervention.

## AUTHOR CONTRIBUTIONS

Jonathan Golledge, Malindu Fernando, and Chanika Alahakoon extracted data; Peter A. Lazzarini, Chanika Alahakoon, and Jaap J. van Netten performed the risk‐of‐bias assessment; Malindu Fernando performed the statistical analysis; Jonathan Golledge wrote the manuscript; All authors provided critical revisions of the manuscript; Jonathan Golledge, Peter A. Lazzarini, and Sicco A. Bus handled funding and supervision.

## CONFLICT OF INTEREST

The author declares that there is no conflict of interest that could be perceived as prejudicing the impartiality of the research reported.

### PEER REVIEW

The peer review history for this article is available at https://publons.com/publon/10.1002/dmrr.3549.

## Supporting information

Supplementary Information S1

## Data Availability

The data that support the findings of this study are openly available in published papers.
