# Supplementary material for: Efficacy of at home monitoring of foot temperature for risk reduction of diabetes‐related foot ulcer: A meta‐analysis
Source: Diabetes Metab Res Rev. 2022 Jun 8;38(6):e3549. doi: 10.1002/dmrr.3549 (PMC9541448; doi:10.1002/dmrr.3549)
Supplement: Supplementary file 1 — Supplementary Information S1 [file DMRR-38-e3549-s001.docx]

# **Supplementary Document**

Table of Contents

[SEARCH STRING 1](#_Toc63959132)

[Supplementary Table 1: Study Characteristics 2](#_Toc63959133)

[Supplementary Table 2: Quality assessment using the Cochrane Tool 5](#_Toc63959134)

[References 6](#_Toc63959135)

# Search string

Medline: exp “diabetic foot” OR exp “diabetic neuropathies” AND randomised controlled trials

Pubmed: “diabetic foot” [MeSH Terms] OR “peripheral neuropathies” [MeSH Terms] AND “temperature” filtered by randomised controlled trials

Cochrane: “diabetic foot syndrome” OR “diabetic foot ulcer” filtered by “trials”

Web of Science: “diabetic foot” searched within by “randomized controlled trials”

# Supplementary Table 1: Study Characteristics

| **Study** | **Sample size** (Screened/ randomised/ completed) | **Number of patients lost for follow up in intervention /control** | **Inclusion and exclusion criteria** | **Intervention** | **Compliance assessment of thermometry** | **Control** | **Primary outcome** | **Other outcomes** |
| --- | --- | --- | --- | --- | --- | --- | --- | --- |
| Armstrong et al. (2007)^1^ | 1942/225/NR | NR | Inclusion: Diabetes type II, Age 18-80, Consenting participants, IWGDF risk category 2 or 3  Exclusion: current diabetic foot complications, severe PAD, cognitive impairment/dementia, substance abuse | Care offered to the control group + infrared thermometry in 6 sites of the foot twice a day. T > 2.2°C difference between either of the sides should be notified to the study coordinator and activity reduced (not specified by how much) | NA | Therapeutic footwear, diabetic foot education and regular foot care. | incidence of foot ulcers | Effect of the intervention on type of ulcer, health-related quality of life, self-efficacy, satisfaction with care, and modulation of activity |
| Lavery et al. (2004)^2^ | NR/85/78 | 4 and 3 | Inclusion: Diabetes type I or II, Age 18-80, Consenting participants, IWGDF risk category 2 or 3  Exclusion: current diabetic foot complications, severe PAD, cognitive impairment/dementia, substance abuse | Care offered to the control group + infrared skin thermometry to measure temperatures on the sole twice daily on 6 sites. If a toe ± metatarsal had been amputated, adjacent anatomic area was measured. T > 2.2°C difference between the left and right corresponding sites should be notified to the nurse case manager and the number of steps reduced in the following days or until temperature difference fell to <2.2°C | NA | Therapeutic footwear, diabetic foot education, and foot evaluation by a podiatrist every 10 –12 weeks. | incidence of foot ulcers | Infections, Charcot's fractures, amputations |
| Lavery et al. (2007)^3^ | 211/173/151* | 10 and 6 (additional 6 dropped out of the 56 who were allocated to structured foot-examination) | Inclusion: Diabetes type I or II, Age 18-80, Consenting participants, previous history of DFU (IWGDF risk category 3), ABPI ≤0.7  Exclusion: current DFD, cognitive impairment | Care offered to the control group + digital infrared thermometry to measure temperatures on each foot on 6 sites daily and record in log book. Training in use before starting via standardised video. T > 2.2 °C between right and left corresponding sites for 2 consecutive days to be notified to the research nurse and activity reduced until the temperature difference normalised | Compliance assessment method was not reported. Compliance rates were 20% in those who developed ulcers and 80% in those who did not in the intervention group | Lower extremity evaluation by a physician every 8 weeks, an education program on foot complications and self-care + footwear | incidence of foot ulcers | Adherence to preventive practices: temperature monitoring, footwear use, contacting the study nurses |
| Skafjeld et al. (2015)^4^ | 110/41/38 | 3 in intervention group | Inclusion: Diabetes type I or II, Age 18-80, Consenting participants, previous history of DFU (IWGDF risk category 3)  Exclusion: current DFD, ABPI <0.7 | Care offered to the control group + digital infrared thermometry to monitor foot temperature at six sites on the foot on daily basis. Recording of daily physical activity using a step-counter during the first week of the study. Temperature >2.2°C difference between corresponding sites on two consecutive days to be notified and reduce activity by 50% until temperature difference normalised | Compliance assessed in a graded scale every 3 months. 67% of the patients indicated they monitored their feet more than 80% of the time. | Daily inspection of feet under, below and between the toes, and daily recording, advise on always wearing their customized footwear, General practitioner care | incidence of foot ulcers | Difference between those who measured T >80% and <80% |
| Bus et al (2021) | 1411/305/304 | 1/ 0 | Inclusion: Diabetes type I or II, Age ≥18, peripheral neuropathy,  history of a  foot ulcer or an amputation in the four years preceding randomization, or past diagnosis of Charcot neuro-osteoarthropathy (i.e. IWGDF risk grade 3);  regular foot care provided by a podiatrist or willingness to undergo such care; and ability to follow study instructions.  Exclusion criteria were: foot ulcer or open amputation site; active Charcot neuroosteoarthropathy;  foot infection; chronic limb-threatening ischemia; wheel-chair bound; bilateral amputation proximal to the tarsometatarsal joint; severe illness that would make 18-  months survival unlikely; or current use of at-home foot temperature monitoring. | Care offered to the control group + infrared skin thermometry to measure skin temperature at six predefined sites daily: hallux, second and third toe, first, third and fifth metatarsal heads. A maximum of two additional plantar foot sites were measured if a previous ulcer or pre-ulcerative lesion (i.e. abundant callus, hemorrhage or blister) had been or was present at another than a predefined site. This selection of measurement sites was different than in previous trials, where only six predefined sites (four at the forefoot, one midfoot and one heel) were used. Participants with a minor or unilateral major amputation that prevented measurement at a predefined site, measured at an adjacent site. Participants recorded each temperature value and the difference between corresponding sites on both feet in a customized form. These forms were returned to the investigator after two weeks at the start, and on a four-weekly basis thereafter.  If the temperature difference at corresponding sites was >2.2°C for two consecutive  days, it was defined as a “hotspot”. Participants were instructed to then substantially  reduce their ambulatory activity, i.e. by at least 50% as judged subjectively, until the  temperature difference normalized to <2.2°C, and to contact their podiatrist for further  instruction and, if needed, treatment. Participants recorded these actions in their  forms. When foot temperature difference exceeded 4°C or did not normalize in two  days, participants were instructed to immediately contact and see their podiatrist.  Participants received mobile-phone text reminders twice every week for the first six  weeks and once every 2 weeks for the remainder of follow-up, to encourage them in  measuring their foot temperatures and in reducing ambulatory activity and contacting  their podiatrist if a hotspot was found. | Ninety-four participants in enhanced therapy (62.3% of total) measured foot  temperature at least 70% of days until a study endpoint. Seventeen participants  (11.2%) never measured foot temperature and 51 (33.8%) did not have a hotspot  during follow-up, as analyzed from returned weekly logs. A total 83 participants  (55.0%) had at least one hotspot during follow-up | Usual care, as provided in the Netherlands according to evidence-based guidelines  consisting of: a) professional foot care and foot screening once every 1-3 months  by a podiatrist; b) therapeutic (custom-made) footwear, if indicated based on ulcer  risk and foot condition; and c) education about self-care practices, ulcer risk factors  and ulcer aetiology. Education was provided via verbal and written information by a  clinician or an investigator at baseline and ad libitum by clinicians during follow-up  clinic visits. All participants were advised to contact their podiatrist if they identified  any area of concern on their foot. | the proportion of participants with a recurrent  foot ulcer in 18 months at a primary site (i.e. the plantar foot, interdigital space or  6  medial, lateral, or anterior forefoot) | Ulcer at any foot site, adherence, serious adverse events and per protocol analyses |
| * Includes a group of patients who underwent structured foot examination as an intervention which is not included in the analysis.  ABPI: Ankle-brachial pressure index  DFU: Diabetes-related foot ulcer  DFD: Diabetes-related foot disease  IWGDF: International working group of diabetic foot (the study inclusion criteria were grouped according to the criteria given by the IWGDF to provide consistency)  IPAQ: International physical activity questionnaire  min: minutes  NA-Not Applicable  NR-Not Reported  PN-Peripheral neuropathy  PAD-Peripheral arterial disease  HbA1C-Haemoglobin A1c level  T-temperature | | | | | | | | |

#

**Supplementary Table 2: Risk of bias assessment using version two of the Cochrane risk-of-bias assessment tool for randomised trials**

| Risk of bias assessment area or question | Trials | | | | |
| --- | --- | --- | --- | --- | --- |
|  | Lavery et al.  (2004)^2^ | Armstrong et al.  (2007)^1^ | Lavery et al.  (2007)^3^ | Skafjeld et al.  (2015)^4^ | Bus et al.  (2021) |
| 1. Bias from randomisation process |  |  |  |  |  |
| 1.1 Was allocation sequence random? | NI | Y | Y | Y | Y |
| 1.2 Was allocation sequence concealed until participants were assigned to intervention/ control? | NI | Y | Y | Y | Y |
| 1.3 Did baseline differences between groups suggest a problem with randomisation? | N | N | N | N | N |
| Risk of bias judgement (low/high/some concerns) | **Some concerns** | **Low** | **Low** | **Low** | **Low** |
| 2. Risk of bias due to deviations from the intended interventions |  |  |  |  |  |
| 2.1 Were participants aware of their assigned intervention during the trial? | Y | Y | Y | Y | Y |
| 2.2 Were carers and people delivering the interventions aware of participants' assigned intervention during the trial? | PN | N | N | Y | Y |
| 2.3. If Y/PY/NI to 2.1 or 2.2:  Were there deviations from the intended intervention that arose because of the trial context? | N | N | N | N | N |
| 2.4 If Y/PY/NI to 2.3: Were these deviations likely to have affected the outcome? | NA | NA | NA | NA | NA |
| 2.5. If Y/PY to 2.4: Were these deviations from intended intervention balanced between groups? | NA | NA | NA | NA | NA |
| 2.6 Was an appropriate analysis used to estimate the effect of assignment to intervention? | Y | N | PN | PY | Y |
| 2.7 If N/PN/NI to 2.6: Was there potential for a substantial impact (on the result) of the failure to analyse participants in the group to which they were randomized? | NA | PN | PN | NA | NA |
| Risk of bias judgement (low/high/some concerns) | **Low** | **Some Concerns** | **Some Concerns** | **Low** | **Low** |
| 3. Risk of bias due to missing outcome data |  |  |  |  |  |
| 3.1 Were data for this outcome available for all, or nearly all, participants randomized? | Y | NI | Y | Y | Y |
| 3.2 If N/PN/NI to 3.1: Is there evidence that the result was not biased by missing outcome data? | NA | PN | NA | NA | NA |
| 3.3 If N/PN to 3.2: Could missingness in the outcome depend on its true value? | NA | PN | NA | NA | NA |
| 3.4 If Y/PY/NI to 3.3: Is it likely that missingness in the outcome depended on its true value? | NA | NA | NA | NA | NA |
| Risk-of-bias judgment (low/high/some concerns) | **Low** | **Low** | **Low** | **Low** | **Low** |
| 4. Bias in measurement of the outcome |  |  |  |  |  |
| 4.1 Was the method of measuring the outcome inappropriate? | N | N | N | N | N |
| 4.2 Could measurement or ascertainment of the outcome have differed between intervention groups? | N | N | N | N | N |
| 4.3 If N/PN/NI to 4.1 and 4.2: Were outcome assessors aware of the intervention received by study participants? | NI | NI | NI | NI | N |
| 4.4 If Y/PY/NI to 4.3: Could assessment of the outcome have been influenced by knowledge of intervention received? | Y | Y | Y | Y | NA |
| 4.5 If Y/PY/NI to 4.4: Is it likely that assessment of the outcome was influenced by knowledge of intervention received? | PY | PN | PY | PN | NA |
| 4. Risk-of-bias judgment (low/high/some concerns) | **High risk** | **Some concerns** | **High risk** | **Some concerns** | **Low** |
| 5. Bias in selection of the reported result |  |  |  |  |  |
| 5.1 Were the data that produced this result analysed in accordance with a prespecified analysis plan that was finalised before unblinded outcome data were available for analysis? | NI | NI | NI | NI | Y |
| Is the numerical result being assessed likely to have been selected, on the basis of the results, from: |  |  |  |  |  |
| 5.2 ... multiple eligible outcome measurements (eg, scales, definitions, time points) within the outcome domain? | N | N | N | N | N |
| 5.3 ... multiple eligible analyses of the data? | N | N | N | N | N |
| Risk-of-bias judgment (low/high/some concerns) | **Some concerns** | **Some concerns** | **Some concerns** | **Some concerns** | **Low** |
| Overall bias Risk-of-bias judgment (low/high/some concerns) | **High Risk** | **High Risk** | **High Risk** | **Some Concerns** | **Low Risk** |
| Comments | High risk as at least one item was rated High Risk | High risk as (>50%) 3 of 5 items were rated some concerns | High risk as at least one item was rated High Risk | Some concerns as (<50%) 2 of 5 items were rated some concerns | Low risk as all items rated low risk |

Y=yes; PY=probably yes; PN=probably no; N=no; NA=not applicable; NI=no information. *Signalling questions for bias due to deviations from intended interventions relate to the effect of assignment to intervention.

#

# References

1. Armstrong DG, Holtz-Neiderer K, Wendel C, Mohler MJ, Kimbriel HR, Lavery LA. Skin temperature monitoring reduces the risk for diabetic foot ulceration in high-risk patients. *American journal of medicine* 2007; **120**(12): 1042‐6.

2. Lavery LA, Higgins KR, Lanctot DR, et al. Home monitoring of foot skin temperatures to prevent ulceration. *Diabetes Care* 2004; **27**(11): 2642-7.

3. Lavery LA, Higgins KR, Lanctot DR, et al. Preventing diabetic foot ulcer recurrence in high-risk patients: use of temperature monitoring as a self-assessment tool. *Diabetes Care* 2007; **30**(1): 14-20.

4. Skafjeld A, Iversen MM, Holme I, Ribu L, Hvaal K, Kilhovd BK. A pilot study testing the feasibility of skin temperature monitoring to reduce recurrent foot ulcers in patients with diabetes--a randomized controlled trial. *BMC Endocrine Disorders* 2015; **15**: 55.
